# Supplementary material for: Ubiquitin proteolysis of a CDK-related kinase regulates titan cell formation and virulence in the fungal pathogen Cryptococcus neoformans
Source: Nat Commun. 2022 Oct 27;13:6397. doi: 10.1038/s41467-022-34151-6 (PMC9613880; doi:10.1038/s41467-022-34151-6)
Supplement: Supplementary file 1 — Supplementary Information [file 41467_2022_34151_MOESM1_ESM.pdf]

Supplementary information

Ubiquitin proteolysis of a CDK-related kinase regulates titan cell formation and virulence in the fungal pathogen *Cryptococcus neoformans*

Chengjun Cao[1], Keyi Wang[2], Yina Wang[1], Tong-Bao Liu[1*], Amariliz Rivera[2], Chaoyang Xue[1,3,4]

[1]Public Health Research Institute, New Jersey Medical School, Rutgers University, Newark, NJ 07103

[2]Center for Immunity and Inflammation, New Jersey Medical School, Rutgers University, Newark, NJ 07103

[3]Department of Microbiology, Biochemistry and Molecular Genetics, New Jersey Medical School, New Jersey Medical School, Rutgers University, Newark, NJ 07103

[4]Rutgers Center for Lipid Research, Rutgers University, New Brunswick, NJ 08901

*Current address: State Key Laboratory of Silkworm Genome Biology, Southwest University, Chongqing 400715, China.

Supplementary Table 1 Strains and plasmids used in this study.

Strains	Genotype	Source/reference
H99	MATalpha	1
KN99**a**	MAT**a**	2
AI187	MAT**a**/alpha	3
CDX36	MATalpha *gpa1::NAT ura5*	4
CDX37	MAT**a** *gpa1::NEO ura5*	4
CDX40	MAT**a** *gpa1::NAT ura5* P_{GPD1}-*GPA1*^{Q284L}*:FLAG-URA*	4
CDX156	MATalpha P_{GPD1}-*GPA1*^{Q284L}	4
CUX1	MAT**a** *crk1::NEO*	This study
CUX2	MATalpha *fbp1::NEO*	5
CUX3	MAT**a** *fbp1::NEO*	5
CUX5	MATalpha *fbp1::NEO FBP1-NAT*	5
CUX6	MAT**a** *fbp1::NEO FBP1-NAT*	5
CUX10	MATalpha *crk1::NEO*	This study
CUX41	MAT**a** *crk1::NEO CRK1-NAT*	This study
CUX63	MATalpha *crk1::NEO CRK1-NAT*	This study
CUX83	MATalpha *ura5*	This study
CUX84	MAT**a** *ura5*	This study
CUX87	MATalpha *fbp1::NEO ura5*	This study
CUX91	MAT**a** *crk1::NEO ura5*	This study
CUX93	MATalpha *crk1::NEO ura5*	This study
CUX118	MATalpha P_{CTR4}-*CRK1:HA-NAT*	This study
CUX119	MATalpha *fbp1::NEO* P_{CTR4}-*CRK1:HA-NAT*	This study
CUX134	MATalpha *fbp1::NEO ura5* P_{ACT1}-*FBP1:FLAG-URA5*	This study
CUX135	MATalpha *fbp1::NEO ura5* P_{ACT1}-*FBP1*$^{\Delta F:}$ *FLAG-URA5*	This study
CUX140	MATalpha *fbp1::NEO* P_{ACT1}-*FBP1:FLAG-URA5* P_{CTR4}-*CRK1:HA-NAT*	This study
CUX141	MATalpha *fbp1::NEO* P_{ACT1}-*FBP1*$^{\Delta F}$*:FLAG-URA5* P_{CTR4}-*CRK1:HA-NAT*	This study
CUX151	MATalpha P_{HIS}-*GFP:CRK1:HA-NAT*	This study
CUX152	MATalpha *fbp1::NEO* P_{HIS}-*GFP:CRK1:HA-NAT*	This study
CUX154	MAT**a** P_{HIS}-*GFP:CRK1:HA-NAT*	This study
CUX191	MATalpha *gpa1::NAT ura5* P_{ACT1}-*CRK1:mCherry*	This study
CUX192	MAT**a** *gpa1::NAT ura5* P_{ACT1}-*CRK1:mCherry*	This study
CUX193	MATalpha *crk1::NEO ura5* P_{GPD1}-*GPA1*^{Q284L}	This study
CUX194	MAT**a** *crk1::NEO ura5* P_{GPD1}-*GPA1*^{Q284L}	This study
CUX221	MATalpha *fbp1::NEO* P_{CRK1}-*CRK1:HA-NAT*	This study
CUX222	MATalpha P_{CRK1}-*CRK1:HA-NAT*	This study
CUX1131	MATalpha P_{CTR4}-*CRK1*$^{\Delta PEST}$*:HA-NAT*	This study
CUX1132	MATalpha *fbp1::NEO* P_{CTR4}-*CRK1*$^{\Delta PEST}$*:HA-NAT*	This study
CUX1135	MATalpha P_{HIS}-*GFP:CRK1:HA-NAT ura5*	This study
CUX1167	MAT**a** *crk1::NEO CRK1-NAT ura5*	This study
CUX1196	MATalpha *ura5* P_{GPD1}-*GPA1:FLAG-URA*	This study
CUX1197	MATalpha *crk1::NEO ura5* P_{GPD1}-*GPA1:FLAG-URA*	This study
CUX1198	MATalpha P_{HIS}-*GFP:CRK1:HA-NAT ura5* P_{GPD1}-*GPA1:FLAG-URA*	This study
CUX1200	MAT**a** *crk1::NEO CRK1-NAT ura5* P_{GPD1}-*GPA1:FLAG-URA*	This study
CUX1205	MATalpha *ura5* P_{GPD1}-*GPA1:FLAG-URA* P_{CTR4}-*CRK1:HA-NAT*	This study
CUX1235	MATalpha *ura5* P_{ACT1}-*GPA1:GFP-URA*	This study
CUX1237	MATalpha *crk1::NEO ura5* P_{ACT1}-*GPA1:GFP-URA*	This study
CUX1238	MAT**a** *crk1::NEO CRK1-NAT ura5* P_{ACT1}-*GPA1:GFP-URA*	This study
CUX1239	MATalpha P_{HIS}-*GFP:CRK1:HA-NAT ura5* P_{GPD1}-*GPA1:FLAG-URA*	This study
CUX1291	MATalpha P_{ACT1}-*CRK1:mCherry-NAT*	This study
CUX1293	MATalpha P_{ACT1}-*CRK1*$^{\Delta PEST}$*:mCherry-NAT*	This study
CUX1309	MATalpha P_{ACT1}-*mCherry-NAT*	This study
CUX1328	MATalpha P_{ACT1}-*CRK1:mCherry-NAT ura5*	This study

CUX1329	MATalpha *P_{ACT1}-CRK1$^{\Delta PEST}$:mCherry-NAT ura5*	This study
CUX1330	MATalpha *P_{ACT1}-CRK1:mCherry-NAT ura5 P_{GPD1}-GPA1:FLAG-URA*	This study
CUX1331	MATalpha *P_{ACT1}-CRK1$^{\Delta PEST}$:mCherry-NAT ura5 P_{GPD1}-GPA1:FLAG-URA*	This study
CUX1340	MATalpha *P_{CTR4}-HA*	
CUX1341	MATalpha *fbp1::NEO ura5 P_{ACT1}-GPA1:GFP-URA*	This study
CUX1342	MATalpha *fbp1::NEO ura5 P_{GPD1}-GPA1:FLAG-URA*	This study

Plasmids	Description	
pCTR4-2	A vector contains the CTR4 inducible promoter	6
pCN19	P_{HIS}-GFP	7
pCXU4	pGADT7+FBP1 (CX34/CX36, BamHI/XhoI)	This study
pCXU24	pGBKT7+CRK1 (CX79/CX33, SfiI/BamHI)	This study
pCXU29	pGBKT7 + CRK1CT (CX90/CX33, EcoRI/BamHI)	This study
pCXU49	pGADT7+FBP1$^{\Delta F}$ (CX34/CX36, BamHI/XhoI)	This study
pCXU83	P_{ACT1}-CRK1:mCherry (CX227/CX230, BamHI/NotI)	This study
pCXU97	pJAF13 + CRK1:HA (CX80/CX298, XbaI/XhoI)	This study
pCXU108	P_{CTR4}-CRK1:HA (CX394/CX395, BamHI)	This study
pCXU117	P_{ACT1}-FBP1$^{\Delta F}$:FLAG (CX225/CX443, BamHI/NotI)	This study
pCXU118	P_{ACT1}-FBP1:FLAG (CX225/CX443, BamHI/NotI)	This study
pCXU160	pCN19 + CRK1:HA(CX394/CX395, BamHI)	This study
pCXU189	pJAF13 + CRK1:HA(CX80/CX298, XbaI/XhoI)	This study
pCXU369	P_{CTR4}-CRK1$^{\Delta PEST}$:HA (CX1871/CX1561, BamHI)	This study
pCXU398	P_{ACT1}-GPA1:GFP (CX1921/CX1924, BamHI/NotI)	This study
pCXU401	P_{ACT1}-CRK1:mCherry (CX1841/CX1314, BamHI)	This study
pCXU402	P_{ACT1}-CRK1 $^{\Delta PEST}$:mCherry (CX1841/CX1314, BamHI)	This study
pDX8	pRCD83 + GPA1:FLAG (JOHE13094/JOHE13095, KpnI + SmaI)	4
pDX121	pGADT7 + GPA1 (JOHE19001/JOHR19002, XmaI/XhoI)	4
pDX122	pGADT7 + GPA1^{Q284L} (JOHE19001/JOHE19002, XmaI/XhoI)	4

Reference

1 Perfect, J. R., Schell, W. A. & Rinaldi, M. G. Uncommon invasive fungal pathogens in the acquired immunodeficiency syndrome. *J Med Vet Mycol* **31**, 175-179 (1993).

2 Nielsen, K. *et al.* Sexual cycle of Cryptococcus neoformans var. grubii and virulence of congenic a and alpha isolates. *Infect Immun* **71**, 4831-4841, doi:10.1128/iai.71.9.4831-4841.2003 (2003).

3 Ianiri, G. & Idnurm, A. Essential gene discovery in the basidiomycete *Cryptococcus neoformans* for antifungal drug target prioritization. *Mbio* **6**, doi:10.1128/mBio.02334-14 (2015).

4 Xue, C., Bahn, Y. S., Cox, G. M. & Heitman, J. G protein-coupled receptor Gpr4 senses amino acids and activates the cAMP-PKA pathway in Cryptococcus neoformans. *Mol Biol Cell* **17**, 667-679, doi:10.1091/mbc.E05-07-0699 (2006).

5 Liu, T. B. *et al.* The F-Box protein Fbp1 regulates sexual reproduction and virulence in Cryptococcus neoformans. *Eukaryot Cell* **10**, 791-802, doi:10.1128/ec.00004-11 (2011).

6 Ory, J. J., Griffith, C. L. & Doering, T. L. An efficiently regulated promoter system for Cryptococcus neoformans utilizing the CTR4 promoter. *Yeast* **21**, 919-926, doi:10.1002/yea.1139 (2004).

7 Price, C. T. *et al.* Molecular mimicry by an F-box effector of Legionella pneumophila hijacks a conserved polyubiquitination machinery within macrophages and protozoa. *Plos Pathog* **5**, e1000704, doi:10.1371/journal.ppat.1000704 (2009).

Supplementary Table 2 Primers used in this study

Primers	Sequences (5'-3')	References/Note
CX5	GTAAAACGACGGCCAG	M13F
CX6	CAGGAAACAGCTATGAC	M13R
CX25	TCTGAGATGGGCGATGCTCTG	CRK1 KO F1
CX26	CTGGCCGTCGTTTTACCATTGCGGAGCCTTTGGAAG	CRK1 KO R1
CX27	GTCATAGCTGTTTCCTGAGGAGAGGGTCAGATCAATCC	CRK1 KO F2
CX28	CATGGTGATGGTAGATCCAAAG	CRK1 KO R2
CX29	ACAGCAGCAGCAGATGTACC	CRK1 KO F3-1
CX30	AAGTGGTCGTCCTCGACGAG	CRK1 KO R3-1
CX31	TCACGCTTCTTTGGCACATCC	CRK1 KO F4
CX34	CATGGGATCCACATGCCCGTACGACCGTCAAG	FBP1 YTH PGADT7 F
CX36	CAGTCTCGAGTCAACGTCCGTTACCGAATC	FBP1 YTH PGADT7 R
CX79	ATGGCCATGGAGGCCATGTCGCAAGACTTTAGCGTCT	CRK1 YTH PGBKT7 F
CX90	AGGCCGAATTCGACAGCCAAGCTTCCTCACAG	CRK1CT YTH PGBKT7 F
CX33	CAGTGGATCCTTATTGTGGAGGATGAGACTGG	CRK1/CRK1CT YTH PGBKT7 R
CX80	CCTCTAGAGCCCCATCAACCCAATCTCACTACCTT	CRK1 Comp / P_{CRK1}-CRK1:HA F
CX81	GCCACAAACTGCCGCCGCTACAT	CRK1 Comp R
CX298	CGTACCTCGAGTTAAGCGTAATCTGGTACGTCGTATGG GTATTGTGGAGGATGAGACTGGTTA	P_{CRK1}-CRK1:HA R
CX82	ATCGTGCGCCAGAAGTCCTCCT	CRK1 KO F3-2
CX83	TCCGCTTCCTTCTTCGCCTTCT	CRK1 KO R3-2
CX198	GAGTTGCGTGATTGTGTTCTTAATTTCACCCCTTT	FBP1 F-box KO R5
CX199	GGTGAAATTAAGAACACAATCACGCAACTCGCCTC	FBP1 F-box KO F5
CX225	CATAAATACAGGATCCATGCCCGTACGACCGTCAAGAA	P_{ACT1}-FBP1:FLAG infusion F1
CX443	ACGCGGCCGCTTACTTATCGTCGTCATCCTTGTAATCAC GTCCGTTACCGAATCGTTGT	P_{ACT1}-FBP1:FLAG infusion R1
CX394	GCAGCCCGGGGGATCCATGTCGCAAGACTTTAGCGTCT	P_{CTR4}-CRK1:HA infusion F1
CX395	TAGAACTAGTGGATCCTTAAGCGTAATCTGGTACGTCG TA	P_{CTR4}-CRK1:HA infusion R1
CX1313	GCAGCCCGGGGGATCCACTGTCGATCACTGCGCT	P_{CRK1}-CRK1:HA F2
CX1314	TAGAACTAGTGGATCCTTGTGGAGGATGAGACTGG	P_{CRK1}-CRK1:HA R2
CX1841	GCAGCCCGGGGGATCCATGTACCCATACGACGTACCAG ATTACGCTATGACCTCGGCGCTCCTATTC	P_{CTR4}-HACRK1 infusion F2
CX1561	TCGTATGGGTAGGATCCTTGTGGAGGATGAGACTGG	P_{CTR4}-CRK1 infusion R2
CX1598	GACGAGCTAGCGTAGATGGAGGTTTTCAACTGGCTCCT AG	CRK1 PEST KO R5
CX1599	TCCATCTACGCTAGCTCGTC	CRK1 PEST KO F5
CX227	CATAAATACAGGATCCATGTCGCAAGACTTTAGCGTCT	CRK1 infusion F
CX228	CGGTACCCGGGGATCCTTATTGTGGAGGATGAGACTGG	CRK1:mCherry overlap R
CX229	CGGGATCCACCGGTCGCCACCATGGACAACACCGAGGA CGTCA	CRK1:mCherry overlap F
CX230	CGGTACCCGGGGATCCTTATCTAGATCCGGTGGATCCC	mCherry fusion R
CX1871	TTCCGGGTGGGATGTACCCATACGACGTACCAGATTAC GCTATGACCTCGGCGCTCCTATTC	HA:CRK1 OE F
CX1561	TCGTATGGGTAGGATCCTTGTGGAGGATGAGACTGG	HA:CRK1 OE R
CX1921	CCAACATGTCTGGATCCATGGGCGGCTGTATGTCTACTC	GPA1 infusion F
CX1922	TGCTCACCATGGGCCCTAAGATACCAGAGTCACGTAA	GPA1:GFP overlap R
CX1923	TAGGGCCCATGGTGAGCAAGGGCGAGGAG	GPA1:GFP overlap F
CX1924	ATTCTTTTACGCGGCCGCCTTGTACAGCTCGTCCATG	GFP infusion R
CX44	GAAATGTCATCGCCTGTGTGCCAA	FBP1 QRT-PCR F
CX45	TGTCAAACTTGATCCTGCGGAGCA	FBP1 QRT-PCR R
CX49	TGAGAAGGACCCTGCCAACA	GAPDH QRT-PCR F
CX50	ACTCCGGCTTGTAGGCATCAA	GAPDH QRT-PCR R
CX382	ACCCGGTAAAACTCAACAGG	PKA1 QRT-PCR F

CX383	GGGTACGGTGGATAAAGATGTC	PKA1 QRT-PCR R
JH12959	TGAGGCTGATGTTCTGAGAG	GPA1 QRT-PCR F
JH12960	GCAACACTTGATCGTACTCTG	GPA1 QRT-PCR R

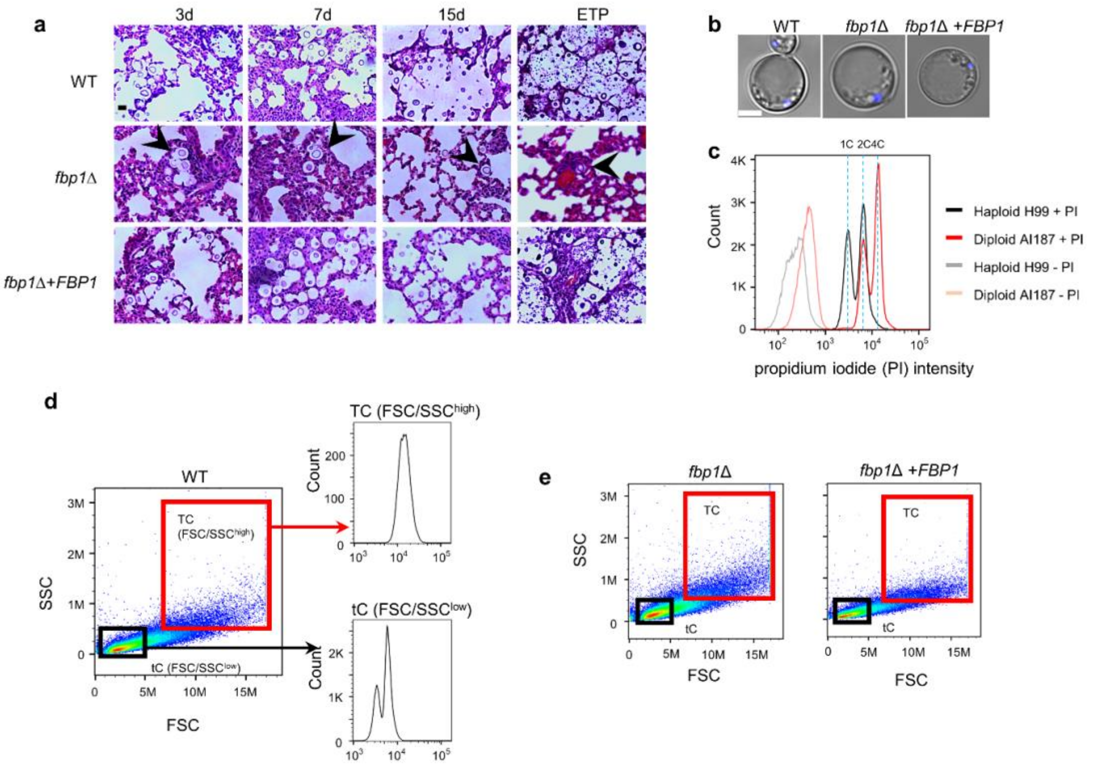

Supplementary Figure 1 Large cells produced by the *fbp1*Δ mutant share the typical characteristics of titan cells. a H&E-stained slides were prepared from cross sections of infected lungs at 3-, 7-, and 15-days post-infection and the end time point (ETP) and visualized by light microscopy. Images are representative of three mice. Arrows indicate yeast cells. Bar, 20 µm. **b** DAPI staining to show the cell nuclei of cells cultured in the in vitro titan cell inducing condition. **c** FACS analysis of the DNA content of haploid H99 and diploid AI187 strains. Cells were fixed and stained by propidium iodide (PI) after overnight culture in YPD. H99 has the standard G1 and G2 cell cycle peaks representing 1C and 2C DNA levels, and the two peaks of AI187 represent 2C and 4C DNA levels. +PI, PI staining; -PI, no PI staining. **d** Cells were cultured under titan cell inducing conditions for 3 days and analyzed by dot plots (FSC/SSC) using flow cytometry. Typical cells and titan cells of WT strains gated through the FSC-A vs SSC-A plot were further shown by the Histograms. The Histograms of typical cells and titan cells were overlaid for comparation as shown in Fig 1e. FSC/SSC^{high} (red border) and FSC/SSC^{low} (black border) represent titan cells (TC) and typical cells (tC), respectively. **e** Cells of the *fbp1*Δ, and *fbp1*Δ+*FBP1* strains were cultured under titan cell inducing conditions for 3 days and analyzed by dot plots (FSC/SSC) using flow cytometry. FSC/SSC^{high} (red border) and FSC/SSC^{low} (black border) represent titan cells (TC) and typical cells (tC), respectively.

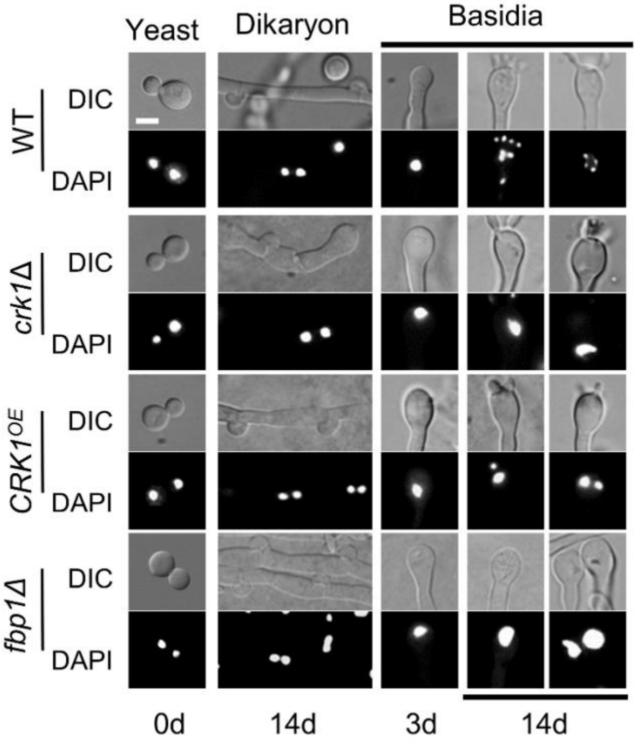

Supplementary Figure 2 Crk1 is required for meiosis and sporulation. Nuclear DAPI staining for yeast cells and mating structures of bilateral mating of the wild type, the *crk1*Δ mutant, the *CRK1*^{OE} strain, and the *fbp1*Δ mutant. Mating structures from bilateral mating cultures at indicated incubation times were visualized under microscopy. DAPI staining was used to track the morphology and location of nuclei in the cells. Images are representative of three independent experiments. Bar, 5 μm.

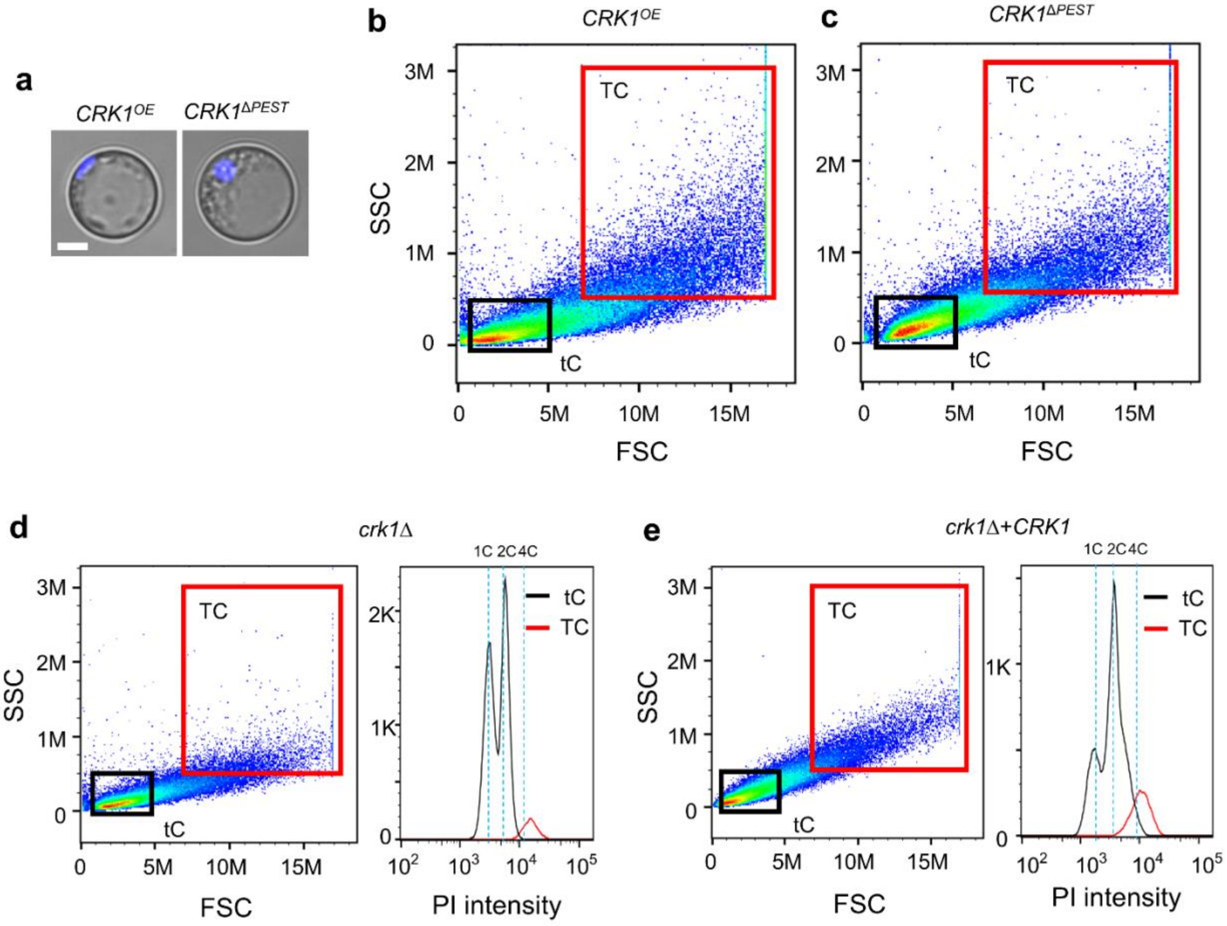

Supplementary Figure 3 The large cells of the *CRK1^{OE}* strain induced under *in vitro* condition are titan cells. a DAPI staining to show the cell nuclei. Images are representative of three independent experiments. Bar, 5 μm. **b-c** The *CRK1^{OE}* (**b**) and the *CRK1$^{\Delta PEST}$* (**c**) strains were cultured under titan cell inducing conditions for 3 days and analyzed by dot plots (FSC/SSC) using flow cytometry. FSC/SSC^{high} (red border) and FSC/SSC^{low} (black border) represent titan cells (TC) and typical cells (tC), respectively. **d-e** FACS analysis of DNA content in the *crk1*Δ mutant (**d**) and the *crk1*Δ+*CRK1* strain (**e**). Cells were cultured under titan cell inducing conditions for 3 days and analyzed by flow cytometry. The population of titan cells showed increased PI fluorescence intensity to >2C.

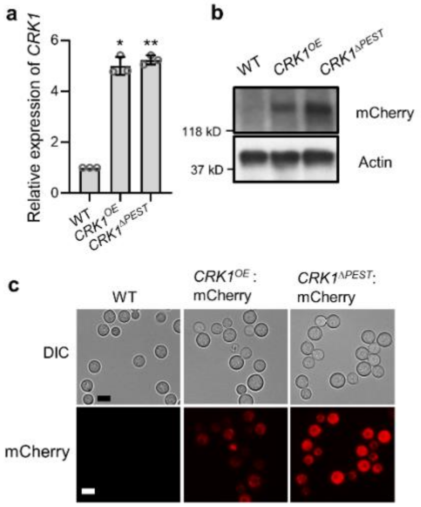

Supplementary Figure 4 Deletion of the PEST domain stabilizes Crk1 protein. a Relative expression levels of *CRK1* in wild type H99, the *CRK1*^{OE} strain, and the *CRK1*$^{\Delta PEST}$ strain under YPD culture condition. The expression level in *wild type* was set as 1. The data shown are cumulated from three independent experiments. Statistical analysis was performed with the two-sided Kruskal-Wallis nonparametric test for multiple comparisons. *, $P = 0.032$. **, $P = 0.002$. **b-c** The expression of Crk1:mCherry or Crk1$^{\Delta PEST}$:mCherry was detected by western blot (**b**) or fluorescence microscopy (**c**). Images are representative of three independent experiments. Bar, 5 μm. Source data are provided as a Source Data file.

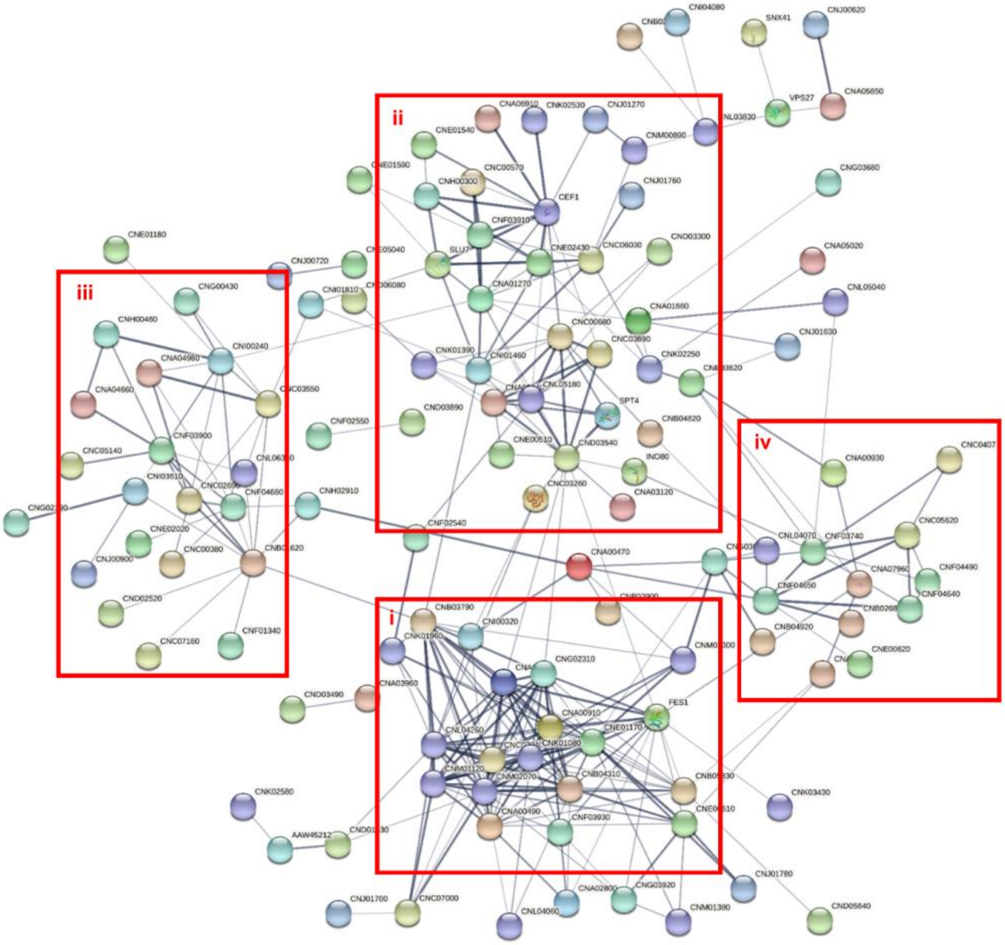

Supplementary Figure 5 STRING analysis to predict interaction network of common DEGs in *fbp1*Δ, *CRK1*^{OE}, and *CRK1*$^{\Delta PEST}$ compared to WT. The STRING (Search Tool for the Retrieval of Interacting Genes/Proteins) program was used to visualize predicted protein-protein interactions for predicted proteins encoded by the 247 common DEGs (http://string-db.org) using the corresponding proteins from *C. neoformans* strain JEC21 in the database. Clusters identified from the network mapping include (i) chaperone and heat-shock proteins involved in protein processing in endoplasmic reticulum, (ii) spliceosome, ribosome, DNA replication and repair proteins involved in pre-mRNA splicing and cell cycle control, (iii) lipid biosynthesis and metabolism, (iv) proteins associated with signaling regulation. Each node represents all the proteins produced by a single protein-coding gene locus. Edges represent protein-protein associations, which indicate proteins jointly contribute to a shared function and do not mean physical interaction.

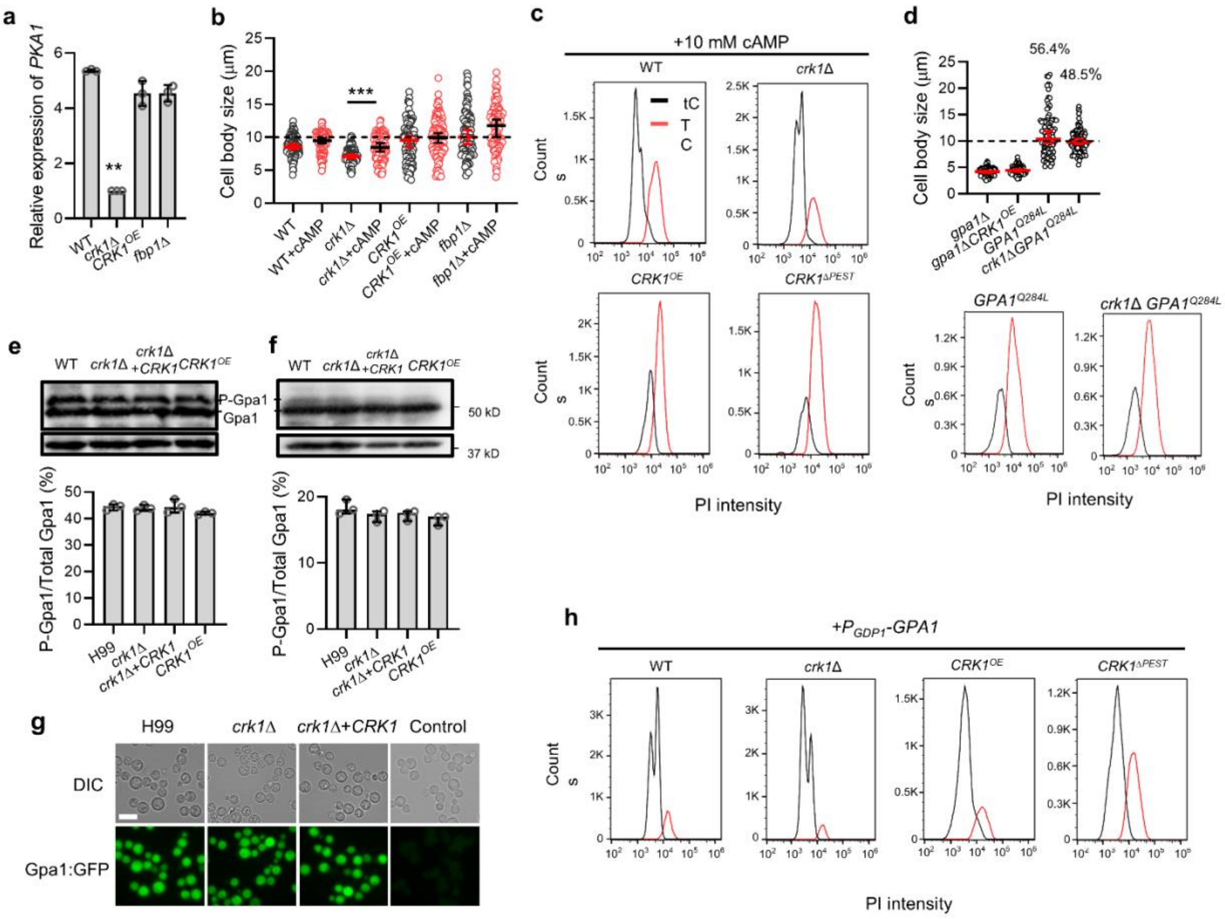

Supplementary Figure 6 Crk1 regulates titan cell formation through the Gpa1-cAMP signaling pathway. a Relative expression levels of *PKA1* in wild type H99, the *crk1*Δ mutant, the *CRK1*^{OE} strain, and the *fbp1*Δ mutant under titan cell inducing conditions. The expression level of *wild type* was set as 1. The data shown are cumulated from three independent experiments. Statistical analysis was performed with the two-sided Kruskal-Wallis nonparametric test for multiple comparisons. **, $P = 0.002$. **b** Cell size under in vitro titan cell inducing conditions in the absence or presence of 10 mM extracellular cAMP. Error bar indicates 95% confidence interval of the median for 100 cells. Statistical analysis was performed based on two-sided Mann-whitney test. ***, $P = 0.0002$. **c** FACS analysis of DNA content following PI staining. Cells were cultured under titan cell inducing conditions in the presence of 10 mM cAMP for 3 days and analyzed by flow cytometry. The population of titan cells showed increased PI fluorescence intensity to >2C. **d** Measurement of cell size of indicated strains after 3 days of incubation in titan cell inducing

conditions and FACS analysis of DNA content of PI-stained cells. Error bar indicates 95% confidence interval of the median for more than 100 cells. **e-f** Gpa1 phosphorylation in nitrogen starvation condition (**e**) and in YPD rich medium (**f**). No significant difference was detected by Kruskal-Wallis nonparametric test for multiple comparisons. Strains expressing Gpa1:FLAG were grown in different conditions and total protein was analyzed by western blotting with FLAG antibody. Error bars indicate the standard deviation of the mean of three independent experiments. **g** The expression of Gpa1:GFP was detected by fluorescence microscopy. Cells without GFP expression were used as a control. Images are representative of three independent experiments. Bar, 5 μm. **h** FACS analysis of DNA content of PI-stained cells. Cells were cultured under titan cell inducing conditions for 3 days and analyzed by flow cytometry. The population of titan cells showed increased PI fluorescence intensity to >2C. Source data are provided as a Source Data file.

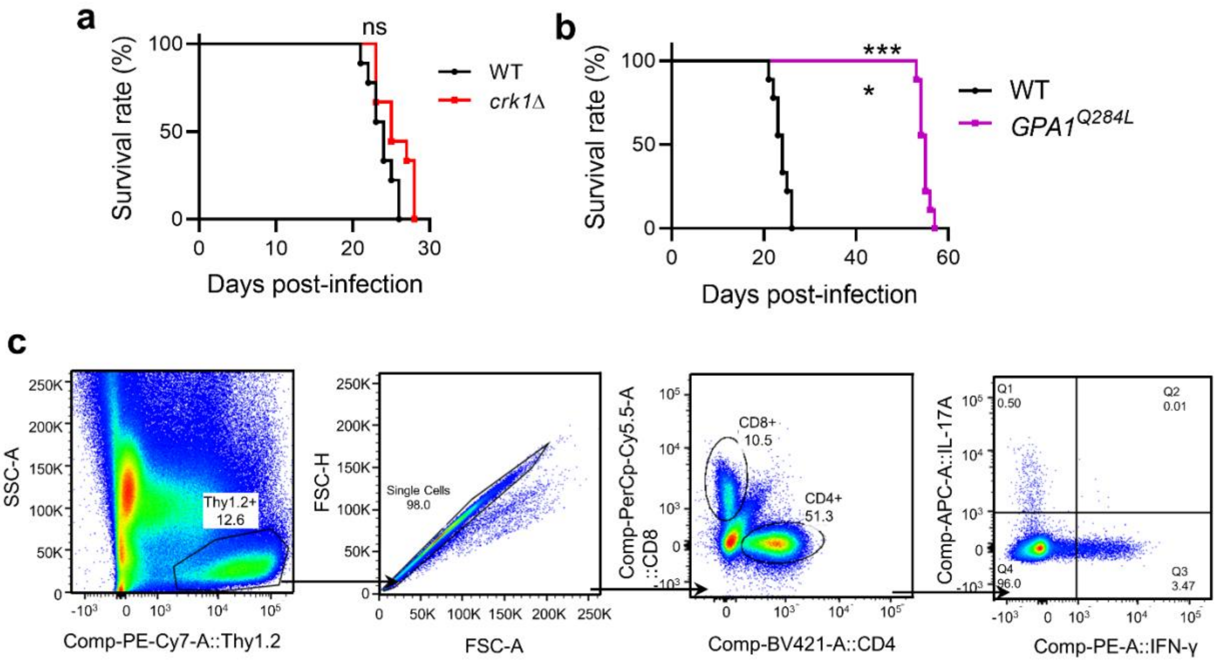

Supplementary Figure 7 Survival curves for mice after intranasal infection. a Female BalB/c mice were intranasally inoculated with 10^5 WT or the *crk1*Δ mutant and their survival rates were plotted against the number of days following inoculation. **b** Survival curves of female BalB/c mice intranasally inoculated with 10^5 cells of the WT or the Gpa1 dominant active allele (*GPA1^{Q284L}*) strain. Statistical analysis was performed based on Log-rank (Mantel-Cox) test. ****, $P < 0.0001$. Source data are provided as a Source Data file. **c** Lymphocytes were gated through the SSC-A Vs Thy1.2, following gate on FSC-A Vs FSC-H to gate on single cells. Then, Thy1.2$^+$ singlets were gated on CD4 Vs CD8 to identify CD4$^+$ T cells and CD8$^+$ T cells. The comparison on cytokine production on CD4$^+$ T cells were further gated on IFN-γ Vs IL-17A.
