# Supplementary material for: Ubiquitin proteolysis of a CDK-related kinase regulates titan cell formation and virulence in the fungal pathogen Cryptococcus neoformans
Source: Nat Commun. 2022 Oct 27;13:6397. doi: 10.1038/s41467-022-34151-6 (PMC9613880; doi:10.1038/s41467-022-34151-6)
Supplement: Supplementary file 3 — Description of Additional Supplementary Files [file 41467_2022_34151_MOESM3_ESM.pdf]

Description of Additional Supplementary Files

Title: Supplementary Data 1.

Description: Analyses of differentially expressed genes in *fbp1*Δ vs WT, $CRK1^{OE}$ vs WT, and $CRK1^{\Delta PEST}$ vs WT in titan cell-inducing conditions.
