# Supplementary material for: Ubiquitin proteolysis of a CDK-related kinase regulates titan cell formation and virulence in the fungal pathogen Cryptococcus neoformans
Source: Nat Commun. 2022 Oct 27;13:6397. doi: 10.1038/s41467-022-34151-6 (PMC9613880; doi:10.1038/s41467-022-34151-6)
Supplement: Supplementary file 5 — Reporting Summary [file 41467_2022_34151_MOESM5_ESM.pdf]

nature portfolio

Corresponding author(s): Chaoyang xue

Last updated by author(s): Oct 9, 2022

Reporting Summary

Nature Portfolio wishes to improve the reproducibility of the work that we publish. This form provides structure for consistency and transparency in reporting. For further information on Nature Portfolio policies, see our Editorial Policies and the Editorial Policy Checklist.

Statistics

For all statistical analyses, confirm that the following items are present in the figure legend, table legend, main text, or Methods section.

n/a	Confirmed	
☐	☒	The exact sample size (*n*) for each experimental group/condition, given as a discrete number and unit of measurement
☐	☒	A statement on whether measurements were taken from distinct samples or whether the same sample was measured repeatedly
☐	☒	The statistical test(s) used AND whether they are one- or two-sided<br>*Only common tests should be described solely by name; describe more complex techniques in the Methods section.*
☒	☐	A description of all covariates tested
☒	☐	A description of any assumptions or corrections, such as tests of normality and adjustment for multiple comparisons
☐	☒	A full description of the statistical parameters including central tendency (e.g. means) or other basic estimates (e.g. regression coefficient) AND variation (e.g. standard deviation) or associated estimates of uncertainty (e.g. confidence intervals)
☐	☒	For null hypothesis testing, the test statistic (e.g. *F*, *t*, *r*) with confidence intervals, effect sizes, degrees of freedom and *P* value noted<br>*Give P values as exact values whenever suitable.*
☒	☐	For Bayesian analysis, information on the choice of priors and Markov chain Monte Carlo settings
☒	☐	For hierarchical and complex designs, identification of the appropriate level for tests and full reporting of outcomes
☐	☒	Estimates of effect sizes (e.g. Cohen's *d*, Pearson's *r*), indicating how they were calculated

Our web collection on statistics for biologists contains articles on many of the points above.

Software and code

Policy information about availability of computer code

Data collection	AriaMx Software version 1.7 for Agilent AriaMx Real-Time PCR System. BD FACSDiva software version 8.0 for BD FACScan flow cytometer and BD LSRFortessa X-20 flow cytometer.
Data analysis	Survival data and other statistical data were analyzed using GraphPad Prism 8.0.2<br>Flow cytometry data were analyzed using Flowjo V10<br>Fluorescence microscopy data and western blotting data were analyzed using Image J 1.53c<br>RNA seq data were performed by RNA-Seq analysis was performed by Novogene according to the company's protocol (https://en.novogene.com). Clean reads were mapped to the annotated genome of C. neoformans H99 using HISAT2 software version 2.0.5.<br>Differential expression analyses were conducted using the DESeq2 package version 1.20.0<br>Clustering analysis of different samples was performed using the R package plots version 3.1.3

For manuscripts utilizing custom algorithms or software that are central to the research but not yet described in published literature, software must be made available to editors and reviewers. We strongly encourage code deposition in a community repository (e.g. GitHub). See the Nature Portfolio guidelines for submitting code & software for further information.

March 2021

Data

Policy information about availability of data

All manuscripts must include a data availability statement. This statement should provide the following information, where applicable:

- Accession codes, unique identifiers, or web links for publicly available datasets
- A description of any restrictions on data availability
- For clinical datasets or third party data, please ensure that the statement adheres to our policy

All raw RNA-seq data were deposited at NCBI Sequence Read Archive (SRA) (https://www.ncbi.nlm.nih.gov/sra) with the accession number PRJNA816899. Processed signature data can be accessed in Supplementary Data 1. The ePESTFind programis available at http://emboss.bioinformatics.nl/cgi-bin/emboss/epestfind. The STRING (Search Tool for Retrieval of Interacting Genes/Proteins) database can be found at http://string-db.org. Source data are provided with this paper.

Field-specific reporting

Please select the one below that is the best fit for your research. If you are not sure, read the appropriate sections before making your selection.

[x] Life sciences [ ] Behavioural & social sciences [ ] Ecological, evolutionary & environmental sciences

For a reference copy of the document with all sections, see nature.com/documents/nr-reporting-summary-flat.pdf

Life sciences study design

All studies must disclose on these points even when the disclosure is negative.

Sample size	Sample size was determined based on the knowledge on good sample size to ensure adequate data for sufficient statistical power and reliable assessments. Sample sizes are always indicated in figure legends or methods section. Sample size was always at least 3 when statistical analysis was required. Sample size of animal was 4 for in vivo titan cells production, 5 for immune response and 10 for survival curve.
Data exclusions	No data was excluded from the analysis.
Replication	All experiments were performed at least two times independently and successfully reproduced. The number of replicates is indicated in the corresponding figure legend and/or in the corresponding material and method section.
Randomization	For all animal studies, animals were randomly assigned to experimental groups
Blinding	Animal studies were blinded during group allocation and experimentation.

Reporting for specific materials, systems and methods

We require information from authors about some types of materials, experimental systems and methods used in many studies. Here, indicate whether each material, system or method listed is relevant to your study. If you are not sure if a list item applies to your research, read the appropriate section before selecting a response.

Materials & experimental systems

n/a	Involved in the study	
[ ]	[x]	Antibodies
[x]	[ ]	Eukaryotic cell lines
[x]	[ ]	Palaeontology and archaeology
[ ]	[x]	Animals and other organisms
[x]	[ ]	Human research participants
[x]	[ ]	Clinical data
[x]	[ ]	Dual use research of concern

Methods

n/a	Involved in the study	
[x]	[ ]	ChIP-seq
[ ]	[x]	Flow cytometry
[x]	[ ]	MRI-based neuroimaging

Antibodies

Antibodies used	For western blotting, rabbit anti-FLAG antibody (Genscript, A01868, 1:2000), mouse anti-FLAG antibody (Genscript, A00187, 1:2000), mouse anti-HA (Genscript, A01244, 1:2000), rabbit anti-FLAG antibody (Genscript, A01963, 1:2000), mouse anti-Actin antibody (Genscript, A00702, 2D1D10, 1:5000), rabbit anti-Actin antibody (Genscript, A01865, 79E2D2, 1:2000), anti-FLAG M2 affinity gel (Sigma aldrich, A2220),anti-mouse (GE Healthcare, NA931V, 1:2000), anti-rabbit (Genscript, A00098, 1:2000). For flow cytometry analysis, antibodies against Thy1.2 (53-2.1 PE-Cy7, BD Biosciences, 561642, 1:200), CD4 (RM4-5 Pacific Blue, BD Biosciences, 558107, 1:200), CD8 (53-6.7 PerCp Cy5.5, BD Biosciences, 561109, 1:200), and IFN-γ (XMG1.2 PE, BD Biosciences, 554412, 1:100), IL-17A (eBio17B7 APC, eBioscience, Inc., 17-7177-81, 1:100).

Validation

rabbit anti-FLAG antibody (Genscript, A01868, 114F12C8, 1:2000). Manufacturer information: GenScript MonoRab™ DYKDDDDK Tag Antibody, mAb, Rabbit specifically recognizes DYKDDDDK tags placed at C-terminal, N-terminal and internal regions of DYKDDDDK fusion proteins.
mouse anti-FLAG antibody (Genscript, A00187, 5A8E5, 1:2000). Manufacturer information: THE™ DYKDDDDK Tag Antibody, mAb, Mouse recognizes C-terminal, N-terminal and internal tagged fusion proteins.
mouse anti-HA (Genscript, A01244, 5E11D8, 1:2000). Manufacturer information: THE™ HA Tag Antibody, mAb, Mouse (A01244) recognizes C-terminal, N-terminal, and internal HA tagged-fusion proteins.
rabbit anti-FLAG antibody (Genscript, A01963, 109B2, 1:2000). Manufacturer information: MonoRab™ HA tag Antibody recognizes HA tags placed at N-terminal, C-terminal, and internal regions of fusion proteins.
mouse anti-Actin antibody (Genscript, A00702, 2D1D10, 1:5000). Manufacturer information: THE™ beta Actin Antibody, mAb, Mouse reacts with mouse, rabbit, chicken, human, hamster, cow, goat, fish, and pig.It has not yet been tested in other species.
rabbit anti-Actin antibody (Genscript, A01865, 79E2D2, 1:2000). Manufacturer information: GenScript MonoRab™ Beta-Actin Antibody, mAb, Rabbit reacts with mouse, human, hamster, and rat. It has not yet been tested in other species.

For flow cytometry analysis,
antibody against Thy1.2 (53-2.1 PE-Cy7, BD Biosciences, 561642, 1:200). Manufacturer information: The 53-2.1 monoclonal antibody specifically binds to the CD90.2 (Thy-1.2) alloantigen on thymocytes, most peripheral T lymphocytes, some intraepithelial T lymphocytes (IEL, DEC), epithelial cells, fibroblasts, neurons, hematopoietic stem cells, but not B lymphocytes, of most mouse strains. The 53-2.1 antibody has been reported not to crossreact with Thy-1.1 (e.g., AKR/J, PL), or with rat Thy-1. CD90 is a glycophosphatidylinositol-anchored membrane glycoprotein of the Ig superfamily that is involved in signal transduction. In addition, there is evidence that CD90 mediates adhesion of thymocytes to thymic stroma. The 53-2.1 antibody has been reported to block the binding of the Rat Anti-Mouse CD90.2 antibody (Clone 30-H12) to immobilized thymocyte membranes.

antibody against CD4 (RM4-5 Pacific Blue, BD Biosciences, 558107, 1:200). Manufacturer information: The RM4-5 monoclonal antibody specifically binds to the CD4 (L3T4) differentiation antigen expressed on most thymocytes, subpopulations of mature T lymphocytes (i.e., MHC class II-restricted T cells, including most T helper cells and immunosuppressive regulatory T cells), and a subset of NK-T cells. CD4 has also been reported to be detected on pluripotent hematopoietic stem cells, bone marrow myeloid and B-lymphocyte precursors, intrathymic lymphoid precursors, and a subset of splenic dendritic cells. CD4 has been reported to be expressed on the plasma membrane of mouse egg cells and is involved in adhesion of the egg to MHC class II-bearing sperm. CD4 is an antigen coreceptor on the T-cell surface which interacts with MHC class II molecules on antigen-presenting cells. It participates in T-cell activation through its association with the T-cell receptor complex and protein tyrosine kinase lck. Purified RM4-5 mAb has been reported to block the binding of FITC-conjugated anti-mouse CD4 clones GK1.5 and H129.19, but not the RM4-4 clone.

antibody against CD8 (53-6.7 PerCp Cy5.5, BD Biosciences, 561109, 1:200). Manufacturer information: The 53-6.7 monoclonal antibody specifically binds to the 38 kDa α and 34 kDa α' chains of the CD8 differentiation antigen (Ly-2 or Lyt-2) of all mouse strains tested. The CD8 α and α' chains (CD8a) form heterodimers with the CD8 β chain (CD8b, Ly-3, or Lyt-3) on the surface of most thymocytes. A subpopulation of mature T lymphocytes (i.e., MHC class I-restricted T cells, including most T suppressor/cytotoxic cells) expresses almost exclusively the CD8 αβ heterodimer. Subsets of γδ TCR-bearing T cells, intestinal intrapithelial lymphocytes, and dendritic cells express CD8a without CD8b. It has been suggested that the expression of the CD8a/CD8b heterodimer is restricted to T lymphocytes which matured in the thymus or in an extrathymic environment that had been influenced by thymus-initiated neuroendocrine signals. CD8 is an antigen coreceptor on the T-cell surface which interacts with MHC class I molecules on antigen-presenting cells or epithelial cells. It participates in T-cell activation through its association with the T-cell receptor complex and protein tyrosine kinase lck (p56 [lck]). The CD8 α and α' chains arise from alternatively spliced messengers of a single CD8a gene. The longer α form associates with p56 [lck] via a CXCP motif in its cytoplasmic domain, which it shares with CD4, but not with CD8b. The truncated α' chain is unable to associate with p56 [lck], and it may function to attenuate the CD8-mediated costimulatory signal during intrathymic T-cell maturation. In vivo and in vitro treatment with 53-6.7 mAb has reportedly been effective at depleting CD8+ peripheral T lymphocytes. The 53-6.7 antibody has also been reported to cross-react with CD8 α- and α'-like polypeptides on subsets of thymic and peripheral lymphocytes in the Egyptian toad, Bufo regularis.

antibody against IFN-γ (XMG1.2 PE, BD Biosciences, 554412, 1:100). Manufacturer information: The XMG1.2 monoclonal antibody specifically binds to mouse interferon-γ (IFN-γ) protein. IFN-γ is a pleiotropic cytokine, of approximately 15-17 kDa, involved in the regulation of inflammatory and immune responses. It plays an important role in activation, growth, and differentiation of T and B lymphocytes, macrophages, NK cells and other non-hematopoietic cell types. IFN-γ production is associated with the Th1 cell differentiation. The purified form of this antibody has been reported to be a neutralizing antibody.

antibody against IL-17A (eBio17B7 APC, eBioscience, Inc., 17-7177-81, 1:100). Manufacturer information: The eBio17B7 antibody reacts with mouse and rat IL-17A with no recognition of IL-17F. Interleukin-17A (IL-17A) is a CD4+ T cell-derived cytokine that promotes inflammatory responses in cell lines and is elevated in rheumatoid arthritis, asthma, multiple sclerosis, psoriasis, and transplant rejection. The cDNA encoding human IL-17A was isolated from a library of CD4+ T cells; the encoded protein exhibits 72 percent amino acid identity with HVS13 , an open reading frame from a T lymphotropic Herpesvirus saimiri, and 63 percent with mouse CTLA-8 (cytotoxic T-lymphocyte associated antigen-8). Human IL-17A exists as glycosylated 20-30 kD homodimers. High levels of IL-17A homodimer are produced by activated peripheral blood CD4+ T-cells. IL-17A enhances expression of the intracellular adhesion molecule-1 (ICAM-1) in human fibroblasts. Human IL-17A also stimulates epithelial, endothelial, or fibroblastic cells to secrete IL-6, IL-8, G-CSF, and PGE2. In the presence of human IL-17A, fibroblasts can sustain the proliferation of CD34+ hematopoietic progenitors and induce maturation into neutrophils. Mouse, rat, and human IL-17A can induce IL-6 secretion in mouse stromal cells, indicating that all homologs can recognize the mouse IL-17A receptor.
IL-23-dependent, IL-17A-producing CD4+ T cells (Th-17 cells) have been identified as a unique subset of Th cells that develops along a pathway that is distinct from the Th1- and Th2- cell differentiation pathways. The hallmark effector molecules of Th1 and Th2 cells, e.g., IFN gamma and IL-4, have each been found to negatively regulate the generation of these Th-17 cells.
Applications Reported: The eBio17B7 antibody has been reported useful for intracellular staining for flow cytometric analysis.

Animals and other organisms

Policy information about studies involving animals; ARRIVE guidelines recommended for reporting animal research

Laboratory animals	C57BL/6 WT and BALB/c WT were purchased from the Jackson laboratory (Bar Harbor, ME). Six-to-eight week-old female mice were used in this study. Mice were housed in groups of five in individually ventilated cages at 21 ± 1°C, 30%-70% relative humidity, 12h/12h dark/light cycle from 7:00am-7:00pm, with free access to food and water and autoclavable mouse houses as environmental enrichment.
Wild animals	No wild animal was used in this study.
Field-collected samples	No field collected samples were used.
Ethics oversight	All animal studies were conducted following biosafety level 2 (BSL-2) protocols and procedures approved by the Institutional Animal Care and Use Committee (IACUC) and Institutional Biosafety Committee of Rutgers University, respectively. The studies were conducted in facilities accredited by the Association for Assessment and Accreditation of Laboratory Animal Care (AAALAC).

Note that full information on the approval of the study protocol must also be provided in the manuscript.

Flow Cytometry

Plots

Confirm that:

- [x] The axis labels state the marker and fluorochrome used (e.g. CD4-FITC).
- [x] The axis scales are clearly visible. Include numbers along axes only for bottom left plot of group (a 'group' is an analysis of identical markers).
- [x] All plots are contour plots with outliers or pseudocolor plots.
- [x] A numerical value for number of cells or percentage (with statistics) is provided.

Methodology

Sample preparation	For DNA content measurement, cells were fixed in ice-cold 70% ethanol overnight at 4°C after 3 days of incubation in titan cell inducing conditions. The fixed cells were washed, resuspended in 0.5 ml of NS buffer (10 mM Tris-HCl, 0.25 M sucrose, 1 mM EDTA, 1 mM MgCl2, 0.1 mM ZnCl2, 0.4 mM phenylmethylsulfonyl fluoride, and 7 mM β-mercaptoethanol). RNase A (0.5 mg/ml) and propidium iodide (10 µg/ml) were added into the suspension and incubated for 2 h at 37°C in the dark. The cells were sonicated for 10 s before analysis with BD FACSVia™ flow cytometer. For intracellular cytokine staining of T cells harvested in BALF, BALF samples of female C57BL/6 mice were harvested at day 7 after infection with 1×106 yeast cells. All collected cells were pelleted and resuspended in 200 µl of RPMI containing 10% fetal calf serum (FCS), penicillin-streptomycin (2,200 U/ml; Gibco), and a gentamicin sulfate solution (1 mg/ml). BALF cells were then plated in a 96-well tissue culture treated plate and restimulated using BD-leukocyte activation cocktail containing BD GolgiPlug (BD Biosciences) according to the manufacturer's instructions. Four hours after activation, BALF cells were surface stained with fluorescently labeled antibodies against Thy1.2, CD4, and CD8. Samples were fixed in 1% paraformaldehyde overnight. Prior to intracellular staining, the samples were permeabilized with 1× BD Perm/Wash buffer according to the manufacturer's instructions. Intracellular cytokine staining (ICCS) was done using fluorescently labeled antibodies against IFN-γ, IL-17A diluted in 1× BD Perm/Wash for 45 min on ice. Samples were immediately washed and analyzed by flow cytometry. For CD4+ T cell isolations, individual samples from each group were pooled (5 mice). CD4+ T cells were purified using a negative-sorting CD4+ isolation kit (Miltenyi Biotec, Inc., Auburn, CA). CD4+ T cell isolation was done by following the manufacturer's instructions and were consistently found to be >90% pure, as assessed by flow cytometry.
Instrument	BD FACSVia™ flow cytometer for DNA content measurement, BD LSRFortessa X-20 flow cytometer for intracellular cytokine analysis.
Software	BD FACSDiva software version 8.0 and FlowJo version 10
Cell population abundance	We recorded at least 100,000 single cells for DNA content measurement and purified 200,000 CD4+ T cells for Cryptococcus-specific CD4+ T cell response. BALF cells were stained with fluorescently labeled antibodies.
Gating strategy	The purpose was to analyze the DNA content of typical cells and titan cells under titan cell inducing conditions. We used FSC to gate typical cells and titan cells. Gating strategies for specific cell populations were illustrated in method "Intracellular cytokine staining of T cells harvested in BALF and flow cytometry". BALFs were cell surface stained for T cells with Thy1.2 (53-2.1 PE-Cy7), CD4 (RM4-5 BV421), CD8 (53-6.7 PerCp Cy5.5) and Intracellular cytokine staining for IFN-γ (XMG1.2 PE) and IL-17A (eBio17B7 APC).

- [x] Tick this box to confirm that a figure exemplifying the gating strategy is provided in the Supplementary Information.
